# Blockade of vascular endothelial growth factor receptors by tivozanib has potential anti-tumour effects on human glioblastoma cells

**DOI:** 10.1038/srep44075

**Published:** 2017-03-13

**Authors:** Majid Momeny, Farima Moghaddaskho, Narges K. Gortany, Hassan Yousefi, Zahra Sabourinejad, Ghazaleh Zarrinrad, Shahab Mirshahvaladi, Haniyeh Eyvani, Farinaz Barghi, Leila Ahmadinia, Mahmoud Ghazi-Khansari, Ahmad R. Dehpour, Saeid Amanpour, Seyyed M. Tavangar, Leila Dardaei, Amir H. Emami, Kamran Alimoghaddam, Ardeshir Ghavamzadeh, Seyed H. Ghaffari

**Affiliations:** 1 Haematology/Oncology and Stem Cell Transplantation Research Centre, Shariati Hospital, School of Medicine, Tehran University of Medical Sciences, Tehran, Iran; 2 Department of Pharmacology, School of Medicine, Tehran University of Medical Sciences, Tehran, Iran; 3 Department of Medical Genetics, School of Medicine, Tehran University of Medical Sciences, Tehran, Iran; 4 Deparmtent of Pathology, Shariati Hospital, School of Medicine, Tehran University of Medical Sciences, Tehran, Iran; 5 Department of Molecular Systems Biology, Cell Science Research Centre, Royan Institute for Stem Cell Biology and Technology, Tehran, Iran; 6 Cancer Biology Research Centre, Tehran University of Medical Sciences, Tehran, Iran; 7 Department of Medicine, Harvard Medical School, Boston, MA, USA; 8 Division of Oncology, Department of Internal Medicine, School of Medicine, Tehran University of Medical Sciences, Tehran, Iran

## Abstract

Glioblastoma (GBM) remains one of the most fatal human malignancies due to its high angiogenic and infiltrative capacities. Even with optimal therapy including surgery, radiotherapy and temozolomide, it is essentially incurable. GBM is among the most neovascularised neoplasms and its malignant progression associates with striking neovascularisation, evidenced by vasoproliferation and endothelial cell hyperplasia. Targeting the pro-angiogenic pathways is therefore a promising anti-glioma strategy. Here we show that tivozanib, a pan-inhibitor of vascular endothelial growth factor (VEGF) receptors, inhibited proliferation of GBM cells through a G2/M cell cycle arrest via inhibition of polo-like kinase 1 (PLK1) signalling pathway and down-modulation of Aurora kinases A and B, cyclin B1 and CDC25C. Moreover, tivozanib decreased adhesive potential of these cells through reduction of intercellular adhesion molecule-1 (ICAM-1) and vascular cell adhesion molecule-1 (VCAM-1). Tivozanib diminished GBM cell invasion through impairing the proteolytic cascade of cathepsin B/urokinase-type plasminogen activator (uPA)/matrix metalloproteinase-2 (MMP-2). Combination of tivozanib with EGFR small molecule inhibitor gefitinib synergistically increased sensitivity to gefitinib. Altogether, these findings suggest that VEGFR blockade by tivozanib has potential anti-glioma effects *in vitro*. Further *in vivo* studies are warranted to explore the anti-tumour activity of tivozanib in combinatorial approaches in GBM.

Gliomas are the most common primary brain tumours with more than 20000 new cases each year in the United States. According to World Health Organization (WHO) prognostic grading system, glial tumours are classified into four grades (grade I–IV), with the most aggressive tumours being grade 4 astrocytomas (also known as glioblastoma; GBM)^1^. GBM has a poor median survival due to its rapid growth, angiogenesis, invasiveness and therapeutic resistance. Treatment of GBM includes maximal surgical resection followed by radiotherapy with concurrent and adjuvant chemotherapy. Regardless of initial response, virtually all patients experience disease relapse^2^. Therefore, there is a pressing need to develop improved therapeutic options for GBM patients.

Angiogenesis, a multi-step process by which tumours develop new vasculature, is a fundamental driver for tumour growth and malignant progression^3^,^4^. The vascular endothelial growth factor (VEGF) pathway is the most promising angiogenic target due to its key roles in angiogenesis and tumour growth. The VEGF family consists of seven ligands including VEGFA, VEGFB, VEGFC, VEGFD, VEGFE, placenta growth factor (PlGF) 1 and PlGF2. The tyrosine kinase receptors in this family include VEGF receptor type 1 (VEGFR1), VEGFR2 and VEGFR3^5^.

There is evidence that enhanced expression of the VEGF family promotes malignant progression and correlates with poor prognosis in GBM^6^,^7^. Extensive endothelial proliferation and vascular permeability leading to vasogenic brain oedema, a major cause of neurologic morbidity, are hallmarks of GBM. This is mainly due to elevated expression of VEGFA and signalling through endothelial VEGFR2^8^,^9^. The degree of VEGFA expression and microvascular density correlate with malignant potential and aggressive behaviour of GBM cells as reflected in disease relapse and overall survival rate^9^,^10^. Consistent with this, blockade of the VEGF pathway has been shown to normalise tumour vessels, improve radiotherapy outcome and extend survival in murine orthotopic models of GBM^11^.

Anti-angiogenic strategies are promising approaches for the treatment of GBM due to the highly vascular nature of these tumours and evidence has determined dependence of glioma growth on tumour-associated angiogenesis^12^,^13^,^14^. GBM patients treated with bevacizumab (anti-VEGFA mAb) alone or in combination with irinotecan chemotherapy have demonstrated improvement in progression-free survival^15^,^16^,^17^. However, lack of improvement in overall survival, frequent development of resistance and incomplete VEGF pathway blockade emphasize the need for more efficacious anti-angiogenic therapies^12^,^18^,^19^. In this regard, an agent with the ability to block all the three VEGF receptors is thought to have improved anti-tumour activity^20^.

Tivozanib (AV-951; AVEO pharmaceuticals) is a pan-VEGFR inhibitor with potential anti-angiogenic and anti-neoplastic activities^21^. Tivozanib has shown anti-tumour activity in xenograft models of prostate, breast, lung, pancreas, glioblastoma and renal cell carcinoma. In both phase I and II clinical trials, it has been found to be well tolerable with manageable side effects and durable clinical activity^22^,^23^. Tivozanib is currently under investigation in a phase II study in patients with recurrent GBM (NCT01846871)^12^. In the present study, we examined the mechanistic activity of tivozanib in human GBM cell lines.

## Results

### Tivozanib inhibits proliferation, clonal growth and anoikis resistance

**Figure 1:**
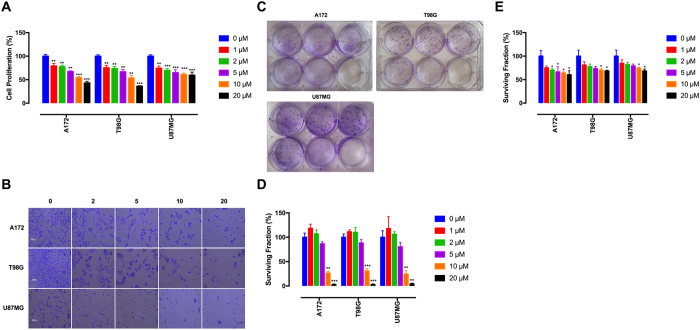
Tivozanib inhibits proliferation, clonal growth and anoikis resistance. (**A**) MTT assay was applied to estimate cell viability after 48 h of treatment with tivozanib. (**B**) The GBM cells were treated with tivozanib for 48 h, stained with crystal violet and imaged by an inverted microscope (images acquired at 10x magnification). (**C,D**) Clonogenic assay was conducted to evaluate the effects of tivozanib on clonal proliferation. (**E**) Anoikis resistance assay was performed with cell culture on poly-HEMA–coated culture dishes for 48 h and the proportion of viable cells was measured by MTT assay. Data are given as mean ± SD, normalized to the untreated control group. Statistically significant values of *p < 0.05, **p < 0.01, and ***p < 0.001 were determined compared with the control.

MTT assay was carried out to determine the effects of tivozanib on proliferation of the GBM cells. Treatment of these cells with tivozanib inhibited their growth (Fig. 1A,B). Moreover, the results of a colony formation assay demonstrate that tivozanib reduced their clonogenic survival (Fig. 1C,D).

Detachment of adherent cells from the extracellular matrix induces anoikis, a special type of apoptosis. Acquisition of resistance to anoikis is a prerequisite for tumour cell survival and spread^24^. Tivozanib diminished anoikis resistance in the GBM cells (Fig. 1E). Taken together, these data illustrate that tivozanib decreased proliferation, clonal growth and anoikis resistance in the GBM cells.

### Tivozanib induces G2/M cell cycle arrest

**Figure 2:**
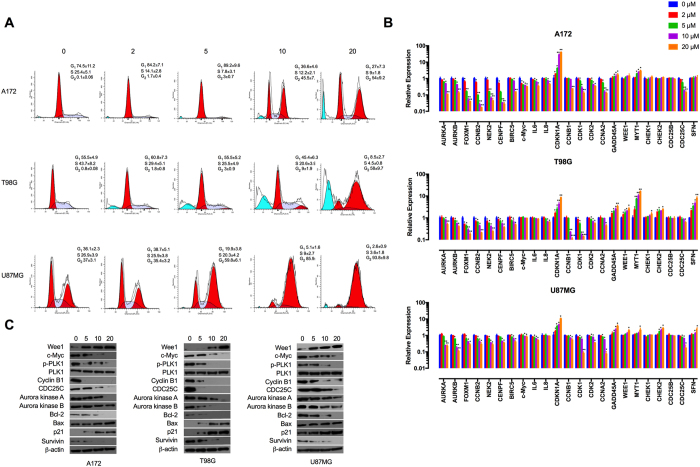
Tivozanib induces G2/M cell cycle arrest. (**A**) Following treatment with tivozanib for 48 h, the cell pellets were fixed and incubated with PI to analyse the cell cycle distribution on a flow cytometer. The graphs are representative of three independent experiments with similar results. (**B**) The cells were treated with tivozanib for 48 h then total RNA was harvested for qRT-PCR analysis. (**C**) Protein lysates from tivozanib-treated cells were subjected to Western blotting and probed with the indicated antibodies. β-actin was used as the loading control. The blots are representative of three independent experiments with similar outcomes. Data are given as mean ± SD. Statistically significant values of *p < 0.05, **p < 0.01, and ***p < 0.001 were determined compared with the control. AURK, aurora kinase; FOXM1, forkhead box M1; CCNB, cyclin B; NEK2, NIMA related kinase 2; CENPF, centromere protein F; IL, interleukin; CDKN1A, cyclin-dependent kinase inhibitor 1 A; CDK, cyclin-dependent kinase; CCNA2, cyclin A2; GADD45A, growth arrest and DNA damage inducible alpha; CHEK, checkpoint kinase; CDC25, cell division cycle 25; MYT1, myelin transcription factor 1; SFN, stratifin.

Due to the anti-proliferative effects of tivozanib, we sought if it inhibits cell cycle progression or affects apoptosis. In A172 and T98G cells, tivozanib increased the percentage of cells in G2/M phase while decreasing the G1/S fraction. Moreover, a small number of cells underwent cell death, as indicated by appearance of a sub-G0/G1 population. Tivozanib-treated U87MG cells displayed an increase in the G2/M population (Fig. 2A).

We next determined the effects of tivozanib on expression of genes and proteins that regulate the G2/M transition. Activation of CDK1/cyclin B complex is a pivotal step in mitotic initiation. WEE1 and myelin transcription factor 1 (MYT1) block mitotic entry via phosphorylation of CDK1, a process reversed by CDC25C^25^,^26^. The phosphatase activity of CDC25C is pre-empted by checkpoint kinase 1 (CHEK1)^27^. Moreover, polo-like kinase 1 (PLK1) drives entry into mitosis via phosphorylation of CDC25C as well as inactivation of WEE1 and MYT1^28^. Both serine/threonine kinases Aurora A and B activate PLK1^29^. Forkhead box protein M1 (encoded by *FOXM1*) is a member of the FOX family of transcription factors that regulates expression of a large array of G2/M-specific genes such as *PLK1*, cyclin B2 (encoded by *CCNB2*), NIMA related kinase 2 (encoded by *NEK2*) and centromere protein F (encoded by *CENPF*)^30^.

Tivozanib inhibited p-PLK1 and decreased expression of Aurora A, Aurora B, CDC25C and cyclin B1 (Fig. 2C, Supplementary Fig. 1). Both mRNA and protein levels of the G2/M checkpoint regulator p21 (encoded by *CDKN1A*) were increased by tivozanib treatment (Fig. 2B,C, Supplementary Fig. 1). In addition, tivozanib reduced the mRNA levels of *FOXM1, CCNB2, NEK2, CENPF, CDK1* and *CDK2* (Fig. 2B). These data suggest that tivozanib inhibited proliferation of the GBM cells through a G2/M cell cycle arrest.

### Tivozanib reduces adhesive and invasive abilities of the GBM cells

**Figure 3:**
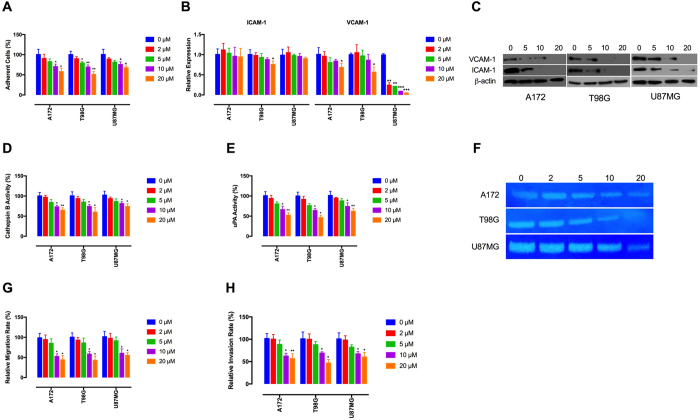
Tivozanib inhibits adhesive and invasive potential of the GBM cells. (**A**) Tivozanib-treated cells were seeded into collagen I-coated culture dishes then the adhesive cells were stained, lysed and the optical densitometry was read. (**B,C**) The effects of tivozanib on mRNA and protein levels of ICAM-1 and VCAM-1 were measured by qRT-PCR and Western blot analysis. β-actin was used as the loading control. The blots are representative of three independent experiments with similar results. (**D**) Equal amounts of secreted protein from treated cells were incubated with a synthetic substrate labelled with amino-4-trifluoromethyl coumarin (AFC). The substrate is cleaved by cathepsin B to release AFC, which is fluorometrically detected. (**E**) The conditioned media from each sample was subjected to a chromogenic substrate, which is cleaved by active uPA and produces a colorimetrically detectable product. **(F)** The conditioned media was collected and separated on a non-reducing polyacrylamide gel containing gelatin A. Gelatinolytic activities are visualized as clear bands against the blue background of stained gelatin. The zymograms are representative of three independent experiments with similar results. The gels were cropped and the full-length gels are presented in Supplementary Fig. 3. **(G)** The cells were placed into 8-μm porous culture inserts, treated with tivozanib and allowed to migrate for 48 h. The migrated cells on the lower surface of the inserts were fixed with methanol, stained with crystal violet, lysed with 30% acetic acid and the optical densitometry was measured at 590 nm. **(H)** For invasion assay, the cells were placed into matrigel-coated inserts and allowed to invade through the matrigel layer for 48 h. Data are given as mean ± SD. Statistically significant values of *p < 0.05, **p < 0.01, and ***p < 0.001 were determined compared with the control.

Adhesion of GBM cells to extracellular matrix (ECM) followed by migration along the ECM and invasion into the adjacent brain tissue is a hallmark of GBM and represents the major obstacle to successful chemo- and surgical therapy^31^. Cell adhesion molecules such as ICAM-1 (intercellular adhesion molecule-1) and VCAM-1 (vascular cell adhesion molecule-1) play key roles in adhesion of GBM cells to the ECM^32^. To investigate whether tivozanib impairs adhesive properties of the GBM cells, we assayed adhesion of tivozanib-treated cells to collagen I which is a substrate for a range of cell adhesion molecules. Tivozanib decreased cell adhesion to collagen I, concomitant with significant reduction of ICAM-1 and VCAM-1 (Fig. 3A–C, Supplementary Fig. 2).

GBM cells secrete proteinases such as cysteine proteinase cathepsin B, serine proteinase urokinase-type plasminogen activator (uPA) and matrix metalloproteinases (MMPs) to remodel the ECM and drive tumour invasion. Cathepsin B stimulates pro-uPA, which leads to proteolytic activation of pro-MMPs and dissolution of the ECM. In this regard, the proteolytic cascade of cathepsin B/uPA/MMP is a potential therapeutic target to restrain GBM invasion^33^. Tivozanib reduced the enzymatic levels of cathepsin B, uPA and MMP-2 (Fig. 3D–F). Moreover, our data demonstrate that tivozanib-mediated inhibition of the proteases was associated with attenuation of cell migration and invasion (Fig. 3G,H).

### Tivozanib enhances sensitivity to gefitinib

Emerging evidence indicates that the VEGF ligands act as survival factors for tumour cells that express the receptors^34^,^35^,^36^. In leukaemia cells, the VEGF family opposes apoptosis via induction of the anti-apoptotic protein Bcl-2 and blockade of VEGFR3 induces chemosensitisation in ovarian cancer cells^37^,^38^. These studies suggest that the VEGF family plays important roles in therapy resistance.

**Figure 4:**
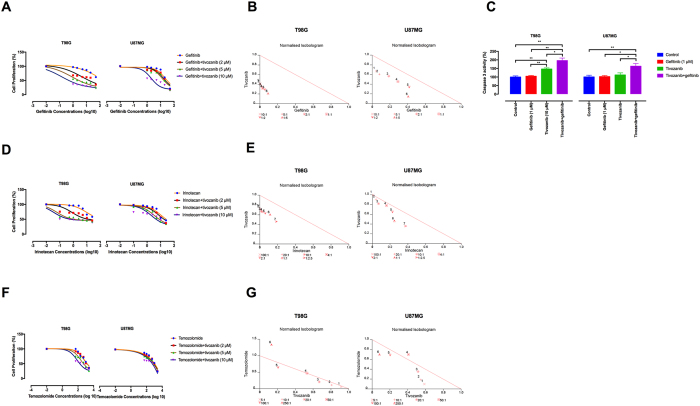
Synergistic activity of tivozanib and gefitinib on cell growth and induction of apoptosis. (**A**) The effect of tivozanib-gefitinib combination therapy on cell proliferation was investigated by MTT assay and shown by EC50 shift analysis. **(B)** Normalised isobolograms of combination of tivozanib (10 μM) and gefitinib (1, 2, 5, 10, 20 and 50 μM). The data were analysed using the CalcuSyn software. The connecting line represents additivity. Data points located below the line indicate a synergistic drug-drug interaction and data points above the line indicate an antagonistic drug-drug interaction. **(C)** The effect of combined tivozanib-gefitinib treatment on activation of caspase 3 was measured by a colorimetric caspase 3 activity assay. **(D)** The effect of tivozanib-irinotecan combination therapy on cell proliferation was investigated by MTT assay and shown by EC50 shift analysis. **(E)** Normalised isobolograms of combination of tivozanib (10 μM) and irinotecan (0.1, 0.5, 1, 2.5, 5, 10 and 25 μg/mL). **(F)** The effect of tivozanib-temozolomide combination therapy on cell proliferation was investigated by MTT assay and shown by EC50 shift analysis. **(G)** Normalised isobolograms of combination of tivozanib (10 μM) and temozolomide (50, 100, 200, 500, 1000 and 2500 μM). Data are given as mean ± SD. Statistically significant values of *p < 0.05 and **p < 0.01 were determined compared with the control.

We thus determined the effects of tivozanib on proliferative response of T98G (temozolomide-resistant) and U87MG (radio- and temozolomide-resistant) cells to gefitinib, irinotecan and temozolomide. The tivozanib-gefitinib combination therapy had synergistic effects on growth inhibition and activation of caspase-3, an indicator of apoptosis (Fig. 4A–C, Table 1). Moreover, tivozanib synergistically increased irinotecan anti-tumour activity in T98G and sensitivity to temozolomide in U87MG cells (Fig. 4D–G, Supplementary Table 1,2). These data suggest that VEGFR blockade by tivozanib enhances chemosensitivity in the GBM cells.

## Discussion

Angiogenesis blockade is considered the most effective targeted therapy for GBM^39^. The anti-glioma effects of anti-angiogenics include inhibition of tumour-associated neoangiogenesis, disruption of tumour stem cell microvascular niche and tumour vascular normalisation^40^,^41^. VEGF pathway inhibitors may also sensitise glioma-associated endothelial cells to cytotoxic therapies^42^,^43^. Moreover, these agents reduce VEGFA-induced brain oedema and thereby, improve chemotherapy delivery to tumour cells^44^,^45^,^46^. Despite this, the direct anti-glial effects of angiogenic inhibitors on VEGFR-expressing GBM cells are largely unknown^47^. In the present study, we investigated the effects of the pan-VEGFR inhibitor tivozanib on the GBM cells.

There is evidence that aberration in cell cycle regulatory network is a frequent event in the pathogenesis of GBM^48^. Alteration of mitotic regulatory proteins such as Aurora kinases A and B, survivin and PLK1 correlates with a dismal survival in GBM^49^,^50^,^51^. Depletion of *AURKA* and *AURKB*, blocking PLK1 and shRNA suppression of *CCNB1* retards tumour growth and increases chemosensitivity^52^,^53^,^54^,^55^. For instance, inhibition of PLK1 in GBM cells inhibits cell proliferation and enhances radiosensitisation^56^. Altogether, these findings imply that the cell cycle regulatory machinery is a potential therapeutic target in GBM in order to interrupt tumour growth^57^,^58^. In agreement, our findings reveal that tivozanib diminished proliferation of the GBM cells through a G2/M cell cycle arrest via up-regulation of p21 and down-modulation of Aurora kinases A and B, cyclin B1 and CDC25C as well as inhibition of p-PLK1.

The initiation, progression and therapeutic resistance of GBM are associated with a subset of tumour cells called glioma-initiating cells (GICs) with an extensive self-renewal capacity^59^,^60^. These glioma stem-like cells (GSCs) have the ability to transdifferentiate into tumour endothelium and contribute to neoangiogenesis and resistance to anti-angiogenics^61^,^62^. Targeted inhibition of signalling networks that drive the self-renewal of GICs is a novel therapeutic strategy in GBM^63^. An autocrine VEGF/VEGFR2 axis is thought to promote the self-renewal capacity and viability of GSCs and its abrogation reduces their survival and tumorigenicity^64^. In addition, the survival of GSCs is strongly dependent on the mitotic regulatory proteins and these cells have been shown to be sensitive to Aurora kinase A and PLK1 inhibitors^65^. Aurora kinase A regulates tumorigenicity of GICs by activating Wnt signalling pathway and its knockdown inhibits their “stemness”, the self-renewal capacity and tumorigenicity^66^. Our present results reveal that tivozanib inhibits p-PLK1 and decreases Aurora kinase A in the GBM cells, suggesting that tivozanib might have anti-tumour activity in GICs cells.

Diffuse infiltration into the normal brain tissue and metastasis throughout the cerebrospinal fluid and leptomeninges are major reasons for tumour recurrence and treatment failure in GBM^67^. The extracellular proteases cathepsin B, uPA and MMP-2 allow the aggressive infiltration by degradation of the ECM components and promoting GBM cell invasion^68^. Elevated expression of cathepsin B correlates with a poor patient survival and increased invasiveness of GBM cells and its depletion interrupts GBM growth, invasion and angiogenesis^69^,^70^,^71^. uPA transcript, protein and enzymatic levels correlate with tumour grade and its inhibition decreases GBM growth and invasion *in vitro* and tumorigenicity *in vivo*^72^,^73^,^74^. In addition, MMP-2 activity is augmented in GBM tumours compared to normal brain and its expression levels is associated with malignant progression^75^. Inhibition of MMP-2 activity or its knockdown reduces GBM invasion^76^,^77^. In ovarian carcinoma cells, the VEGF/VEGFR loop promotes tumour invasion through induction of uPA and MMP-2, suggesting that blockade of the VEGF family might hinder tumour invasiveness^78^. In concert with this, the results of the current study demonstrate that tivozanib decreased invasive potential of the GBM cells via inhibition of the enzymatic levels of cathepsin B, uPA and MMP-2. These finding further suggest that tivozanib could be applied in combination with radio- and chemotherapies in order to halt intrinsic and post-treatment invasion of GBM.

Amplification and overexpression of EGFR are observed in 50% of gliomas and associate with a worse prognosis^79^. Despite this, clinical trials with EGFR-targeted therapies have not shown marked activity in GBM patients^80^,^81^. Evidence indicates that increased tumour angiogenesis drives resistance to anti-EGFR therapies^82^,^83^. Elevated expression of VEGFA contributes to development of gefitinib-resistant colon cancer cells, which was abrogated by treatment with a VEGFR2 inhibitor^84^. Our findings suggest that tivozanib potentiates anti-tumour activity of gefitinib in the GBM cells.

Members of the Bcl-2 family of proteins play central roles in treatment response in GBM patients^85^. High levels of *BAX* correlate with better clinical outcome^86^. ABT737, a Bcl-2 family inhibitor, sensitises GBM cells to both anti-cancer drugs and the death ligand TRAIL^87^. In addition, the inhibitor of apoptosis protein survivin (encoded by *BIRC5*) has been shown to mediate radioresistance in GBM cells^88^. These findings suggest that targeting the apoptotic machinery enhances GBM responsiveness to pro-apoptotic agents^89^. Consistent with this, our data demonstrate that tivozanib-induced sensitisation to gefitinib might be through down-regulation of the anti-apoptotic proteins survivin and Bcl-2.

**Figure 5:**
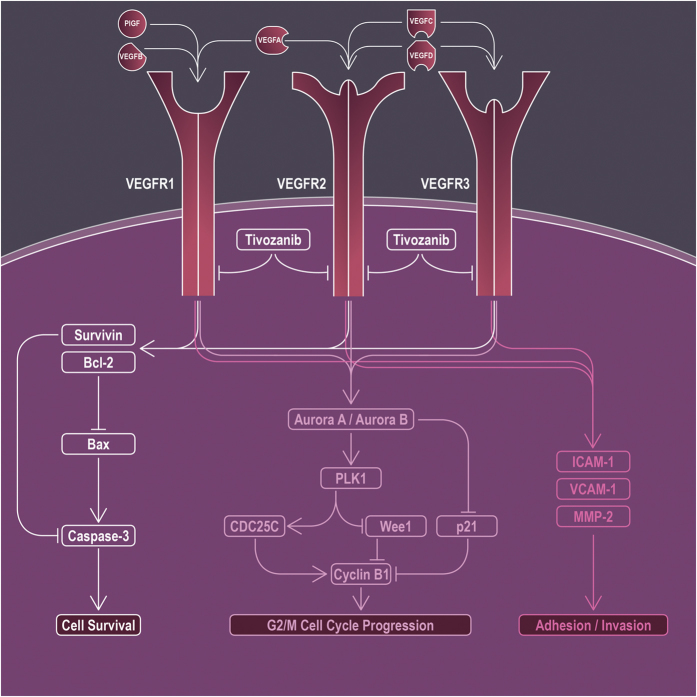
A schematic representation of the anti-tumour effects of tivozanib on the GBM cells. Tivozanib induced a G2/M cell cycle arrest through up-regulation of p21 and suppression of Aurora kinase A, Aurora kinase B, p-PLK1, cyclin B1 and CDC25C. Tivozanib decreased adhesive and invasive potential of the GBM cells via inhibition of ICAM-1, VCAM-1 and enzymatic levels of MMP-2. Furthermore, tivozanib increased anti-tumour activity of gefitinib on cell growth and induction of apoptosis through down-regulation of the anti-apoptotic proteins survivin and Bcl-2.

Taken together, our data demonstrate that inhibition of VEGF receptors by tivozanib reduced proliferative and invasive characteristics of the GBM cells and provide new insight into the mechanistic activity of tivozanib (Fig. 5). Combination of tivozanib with gefitinib synergistically increased the anti-tumour activity of gefitinib on cell growth and induction of apoptosis. These findings suggest that tivozanib has potential anti-glioma effects. Further *in vivo* studies are warranted to elucidate the anti-tumour activity of tivozanib in combinatorial approaches in GBM.

## Materials and Methods

### Antibodies and chemicals

Antibodies were obtained as follows: p-PLK1 (Thr210) (Cell Signalling Technology); Aurora A (Millipore); Aurora B (Abcam); PLK1 (clone F-8), VCAM-1 (clone H-276), ICAM-1 (clone H-108), Bcl-2 (clone N-19), Bax (clone N-20), survivin (clone FL-142), p21 (clone C-19), cyclin B1 (clone GNS1), Wee1 (clone C-20), CDC25 (clone C-20), c-Myc (clone 9E10) and β-actin (Santa Cruz Biotechnology).

Tivozanib was purchased from AdooQ BioScience (Irvine, CA, USA). Gefitinib (EGFR small molecule inhibitor) and temozolomide (a DNA alkylating agent) were obtained from ChemieTek (Indianapolis, IN, USA). Irinotecan (a topoisomerase I inhibitor) was purchased from pharmacy of Shariati hospital (Tehran, Iran). Poly-hydroxyethylmethacrylate polymer (poly-HEMA) was obtained from Santa Cruz Biotechnology.

### Human glioblastoma cell lines

Human glioblastoma cell lines A172, T98G and U87MG were obtained from National Cell Bank of Iran (NCBI, Tehran, Iran). All the cell lines were authenticated by STR profiling using Cell ID^TM^ system (Promega) and were routinely checked for mycoplasma infection. Cell cultures were maintained at 37 °C in 5% CO_2_ in a humidified incubator and cultured according to NCBI recommendations.

### Cell proliferation

The cells in logarithmic growth phase were plated in 96-well plates. After incubation at 37 °C for 24 h, the cultures were exposed to desired concentrations of the drugs for 48 h and the proportion of viable cells was determined by MTT assay. Synergism was determined by calculation of combination index (CI) according to Chou and Talalay^90^ using CalcuSyn software (Biosoft, Cambridge, UK). CI < 1, CI = 1, and CI > 1 represent synergism, additive effects and antagonism of two drugs, respectively.

### Crystal violet staining

The cells were plated at a density of 6 × 10^4^ cells in 6-well plates and treated with tivozanib for 48 h. The cultures were fixed with ice-cold methanol and stained with crystal violet (0.5% w/v). The images were acquired with an inverted microscope.

### Colony formation assay

Cells were seeded onto 6-well plates with a density of 1000–2000 cell/well. After 12 h, the cells were treated with tivozanib for 48 h. The media was then changed to drug-free media and the cells were incubated at 37 °C in 5% CO_2_ for 10 d. The cultures were fixed in ice-cold methanol and stained with crystal violet solution (1% w/v). The colonies were counted and surviving fraction (SF) was estimated as: (mean colony counts)/(cells plated) × (plating efficiency), where plating efficiency (PE) was determined as (mean colony counts)/(cells plated for untreated controls)^91^.

### Anoikis resistance assay

To mimic anchorage-independent growth conditions, culture dishes were coated with poly-HEMA, as described by Sher *et al*.^92^. Approximately 5 × 10^3^ cells were seeded under anchorage-independent conditions with tivozanib treatment for 48 h. Cell viability was determined by MTT assay.

### Analysis of gene expression by quantitative reverse transcription-PCR

The quantitative reverse transcription-PCR (qRT-PCR) analysis was performed on a LightCycler^®^ 96 instrument (Roche Molecular Diagnostics) using RealQ SYBR Green PCR reagents (Ampliqon, Copenhagen, Denmark). The primers used are listed in Table 2. Expression of target mRNA was normalized to the expression of beta-2-microglobulin (*B2M*) for generation of ΔC_T_ values and relative mRNA expression was quantified using the ΔΔC_T_ method, where C_T_ is cycle threshold^93^.

### Western blot analysis

The cells were lysed for 30 min in ice-cold RIPA buffer (50 mM Tris-HCl, pH 8.0, 150 mM NaCl, 1.0% NP-40, 0.5% sodium deoxycholate and 0.1% SDS) containing protease and phosphatase inhibitors (Roche Molecular Biochemicals). Equal amounts of protein (50 μg) were separated on SDS-PAGE, transferred to PVDF membrane (Membrane Solutions, TX, USA) then probed with primary and horseradish peroxidase (HRP)-conjugated secondary antibodies (Sigma). β-actin was used as the loading control and proteins were detected using a BM chemiluminescence detection kit (Roche Molecular Biochemicals).

### Cell cycle analysis

Cell cycle analysis was performed using propidium iodide (PI) staining. Harvested cells were washed in ice-cold PBS, fixed in 70% ethanol and stored at −20 °C overnight. The cell pellets were then incubated with RNase A (100 μg/mL) (Sigma), PI (50 μg/mL) (Sigma) and 0.05% Triton X-100. Cellular DNA content was analysed on a FACSCalibur (BD Bioscience) flow cytometer equipped with CellQuest Pro software.

### Zymography

Equal amounts of protein from the supernatants were applied to polyacrylamide gels copolymerized with gelatin A (Sigma). After electrophoresis, the gels were rinsed in re-activation buffer overnight and then stained with Coomassie Brilliant Blue. Areas of enzymatic activity appeared as clear bands over the dark background^94^.

### Urokinase-type plasminogen activator activity assay

Urokinase-type plasminogen activator (uPA) activity was assayed with a uPA-specific chromogenic substrate according to the manufacturer’s instructions (Millipore). Equal amounts of protein from the conditioned media were added to the chromogenic substrate and incubated at 37 °C for 1 h. The samples were read at 405 nm.

### Cathepsin B activity assay

To monitor the effect of tivozanib on activity of secreted cathepsin B, a cathepsin B activity assay (Abcam) was applied following the manufacturer’s instructions. Briefly, equal amounts of protein from the supernatants were incubated with a specific substrate of cathepsin B at 37 °C for 2 h. The substrate contains amino-4-trifluoromethyl coumarin, which is released because of cathepsin B activity. The samples were then read in a Synergy HT fluorescent microplate reader (BioTek Instruments) with a 400 nm excitation filter and a 505 nm emission filter. The cathepsin B activity was obtained by comparing the relative fluorescence units of the samples with the control group.

### Cell adhesion

Cell adhesion assay was carried out as described by Ueno *et al*.^95^. The cells were treated with tivozanib for 48 h and then seeded in collagen I-coated 60 mm dishes (Biocoat Cell Environments; Becton Dickinson). After incubation for 15 min at 37 °C, cells were washed twice with cold PBS, stained with 1% crystal violet, lysed with 30% acetic acid and the optical densitometry was measured at 590 nm.

### Cell migration and invasion

Transwell cell migration and invasion assays were carried out as described earlier^96^.

### Caspase 3 activity assay

A colorimetric caspase 3 activity assay (Sigma) was conducted to quantitatively determine apoptotic cell death. Cell lysates from both adherent and floating cells were centrifuged at 20000 × g for 10 min. Twenty μg of the supernatant was incubated with 85 μL of assay buffer plus 10 μL of caspase 3 substrate acetyl-Asp- Glu-Val-Asp p-nitroanilide (Ac-DEVD-pNA) in a 96-well plate at 37 °C for 12 h. The samples were then read at 405 nm in an ELISA reader.

### Statistical analysis

All data were evaluated in triplicate against untreated control cells and collected from three independent experiments. Data were graphed and analysed by GraphPad Prism Software 7.0a using one-way ANOVA and the unpaired two-tailed Student’s *t* test. All data are presented as mean ± standard deviation (SD).

## Additional Information

**How to cite this article:** Momeny, M. *et al*. Blockade of vascular endothelial growth factor receptors by tivozanib has potential anti-tumour effects on human glioblastoma cells. *Sci. Rep.* **7**, 44075; doi: 10.1038/srep44075 (2017).

**Publisher's note:** Springer Nature remains neutral with regard to jurisdictional claims in published maps and institutional affiliations.

## Supplementary Material

Supplementary Information

## Figures and Tables

**Table 1:** Combination index (CI) and dose reduction index (DRI) of tivozanib and gefitinib combination in T98G and U87MG cells.

Concentrations (μM)		fa	T98G	CI	DRI	
Tivozanib	Gefitinib				Tivozanib	Gefitinib
10	1	0.64		0.43	2.38	212.76
10	2	0.65		0.41	2.45	113.87
10	5	0.68		0.37	2.84	54.38
10	10	0.7		0.35	3.18	31.78
10	20	0.72		0.35	3.43	17.68
10	50	0.78		0.31	4.69	10.86
**U87MG**						
10	1	0.43		0.72	1.49	21.99
10	2	0.47		0.67	1.65	12.4
10	5	0.49		0.75	1.76	5.35
10	10	0.57		0.74	2.2	3.44
10	20	0.66		0.76	2.95	2.4
10	50	0.86		0.55	6.73	2.47

DRI represents the order of magnitude of dose reduction that is allowed in combination for a given degree of effect as compared with the dose of each drug alone. “fa” denotes fraction affected.

**Table 2:** Nucleotide sequences of the primers used for qRT-PCR.

Gene	Accession	Forward Primer	Reverse Primer	Amplicon
*B2M*	NM_004048	GATGAGTATGCCTGCCGTGT	CTGCTTACATGTCTCGATCCCA	79
*CDKN1A*	NM_000389	CCTGTCACTGTCTTGTACCCT	GCGTTTGGAGTGGTAGAAATCT	130
*c-MYC*	NM_002467	GTCAAGAGGCGAACACACAAC	TTGGACGGACAGGATGTATGC	162
*BIRC5*	NM_001168	CCAGATGACGACCCCATAGAG	TTGTTGGTTTCCTTTGCAATTTT	152
*IL6*	NM_000600	ACTCACCTCTTCAGAACGAATTG	CCATCTTTGGAAGGTTCAGGTTG	149
*IL8*	NM_000584	GCTCTGTGTGAAGGTGCAGTT	ACCCAGTTTTCCTTGGGGTC	203
*CCNB1*	NM_031966	AATAAGGCGAAGATCAACATGGC	TTTGTTACCAATGTCCCCAAGAG	111
*CDK1*	NM_001786	AAACTACAGGTCAAGTGGTAGCC	TCCTGCATAAGCACATCCTGA	148
*CDK2*	NM_001798	CCAGGAGTTACTTCTATGCCTGA	TTCATCCAGGGGAGGTACAAC	90
*CCNA2*	NM_001237	TGGAAAGCAAACAGTAAACAGCC	GGGCATCTTCACGCTCTATTT	109
*WEE1*	NM_003390	AGGGAATTTGATGTGCGACAG	CTTCAAGCTCATAATCACTGGCT	160
*GADD45A*	NM_001924	GAGAGCAGAAGACCGAAAGGA	CACAACACCACGTTATCGGG	145
*CHEK1*	NM_001114122	ATATGAAGCGTGCCGTAGACT	TGCCTATGTCTGGCTCTATTCTG	183
*CHEK2*	NM_007194	TCTCGGGAGTCGGATGTTGAG	CCTGAGTGGACACTGTCTCTAA	205
*CDC25B*	NM_021873	GGCTGAGGAACCTAAAGCCC	CTTTCCGTCTACTGTCTGTAGGA	139
*CDC25C*	NM_001790	TCTACGGAACTCTTCTCATCCAC	TCCAGGAGCAGGTTTAACATTTT	98
*SFN*	NM_006142	TGACGACAAGAAGCGCATCAT	GTAGTGGAAGACGGAAAAGTTCA	133
*MYT1*	NM_004535	CGCCTCTGTTTCGGATGAATC	TGAATCTCGTCCTGTCCTGAC	75
*ICAM1*	NM_000201	AGCTTCGTGTCCTGTATGGC	TTTTCTGGCCACGTCCAGTT	70
*VCAM-1*	NM_001078	TGTTTGCAGCTTCTCAAGCTTTT	GATGTGGTCCCCTCATTCGT	181
*CCNB2*	NM_004701	CTACAGCGTCGAAGATCCCC	CAAATCACTGGACACCGTCG	172
*FOXM1*	NM_202002	ATAGCAAGCGAGTCCGCATT	AGCAGCACTGATAAACAAAGAAAGA	151
*CENPF*	NM_016343	GCTGCGGGCAGTTTGAATTAG	AAATAAACTTGCTCTCGGGGACG	105
*NEK2*	NM_002497	GTCTCTGGCAAGTAATCCAGAACT	CTTCAGGTCCTTGCACTTGG	151
*AURKA*	NM_198433	GGATATCTCAGTGGCGGACG	GCAATGGAGTGAGACCCTCT	211
*AURKB*	NM_004217	GCTCTCCTCCCCCTTTCTCT	TGTGAAGTGCCGCGTTAAGA	245
