# Supplementary material for: Blockade of vascular endothelial growth factor receptors by tivozanib has potential anti-tumour effects on human glioblastoma cells
Source: Sci Rep. 2017 Mar 13;7:44075. doi: 10.1038/srep44075 (PMC5347040; doi:10.1038/srep44075)
Supplement: Supplementary Information [file srep44075-s1.pdf]

Blockade of vascular endothelial growth factor receptors by tivozanib has potential anti-tumour effects on human glioblastoma cells

Majid Momeny[1,+], Farima Moghaddaskho[1,+], Narges K. Gortany[2], Hassan Yousefi[3], Zahra Sabourinejad[4], Ghazaleh Zarrinrad[1], Shahab Mirshahvaladi[5], Haniyeh Eyvani[1], Farinaz Barghi[1], Leila Ahmadinia[1], Mahmoud Ghazi-Khansari[2], Ahmad R. Dehpour[2], Saeid Amanpour[6], Seyyed M. Tavangar[4], Leila Dardaei[7], Amir H. Emami[8], Kamran Alimoghaddam[1], Ardeshir Ghavamzadeh[1], Seyed H. Ghaffari[1,*]

[1]Haematology/Oncology and Stem Cell Transplantation Research Centre, Shariati Hospital, School of Medicine, Tehran University of Medical Sciences, Tehran, Iran
[2]Department of Pharmacology, School of Medicine, Tehran University of Medical Sciences, Tehran, Iran
[3]Department of Medical Genetics, School of Medicine, Tehran University of Medical Sciences, Tehran, Iran
[4]Deparmtent of Pathology, Shariati Hospital, School of Medicine, Tehran University of Medical Sciences, Tehran, Iran
[5]Department of Molecular Systems Biology, Cell Science Research Centre, Royan Institute for Stem Cell Biology and Technology, Tehran, Iran
[6]Cancer Biology Research Centre, School of Medicine, Tehran University of Medical Sciences, Tehran, Iran
[7]Department of Medicine, Harvard Medical School, Boston, MA, USA
[8]Division of Oncology, Department of Internal Medicine, School of Medicine, Tehran University of Medical Sciences, Tehran, Iran

[+]These authors contributed equally as first authors.

*Correspondence to: Seyed H. Ghaffari, email: shghaffari200@yahoo.com

Keywords: Glioblastoma; VEGF family; Tivozanib; Gefitinib

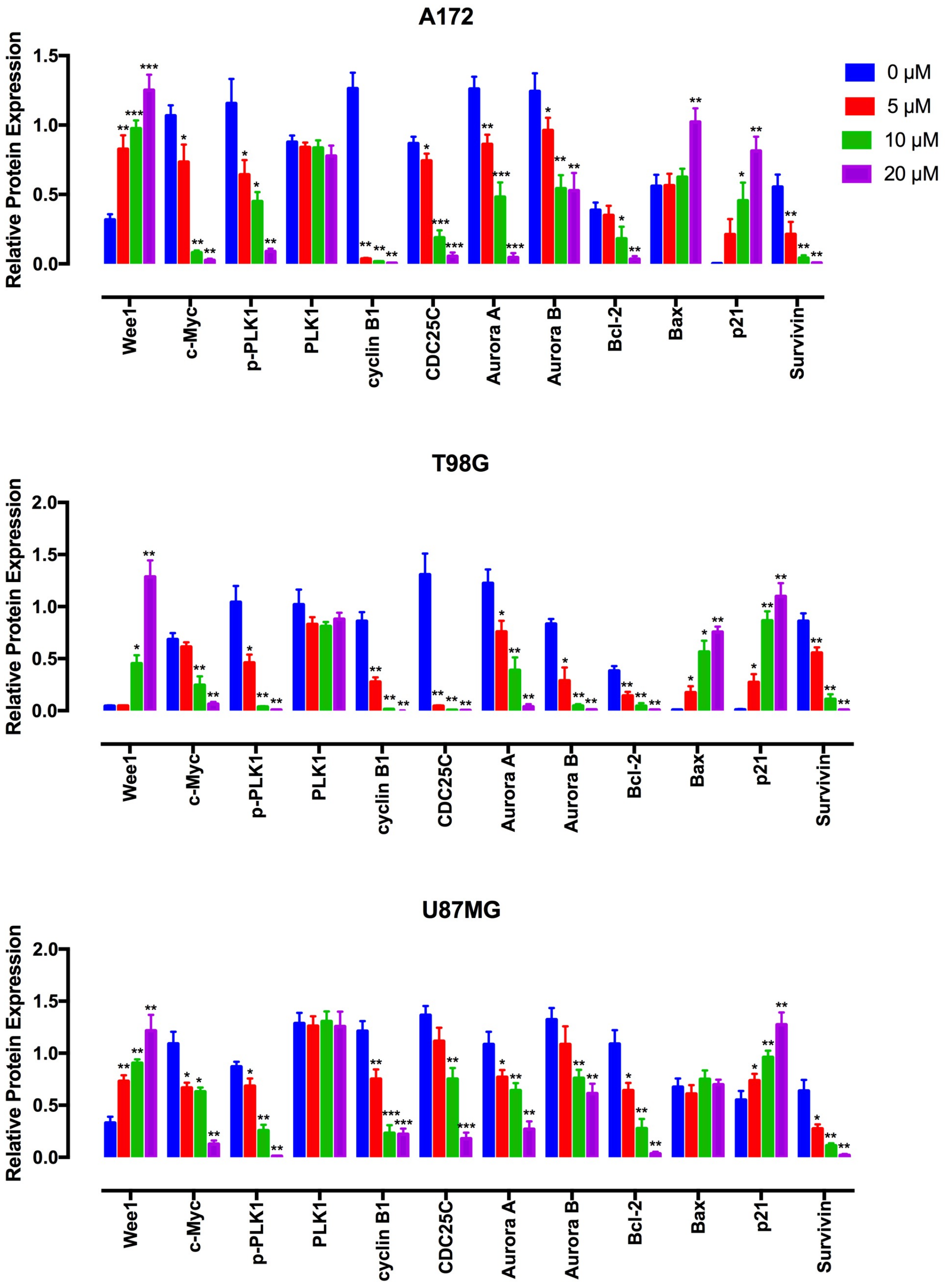

Supplementary Fig. 1

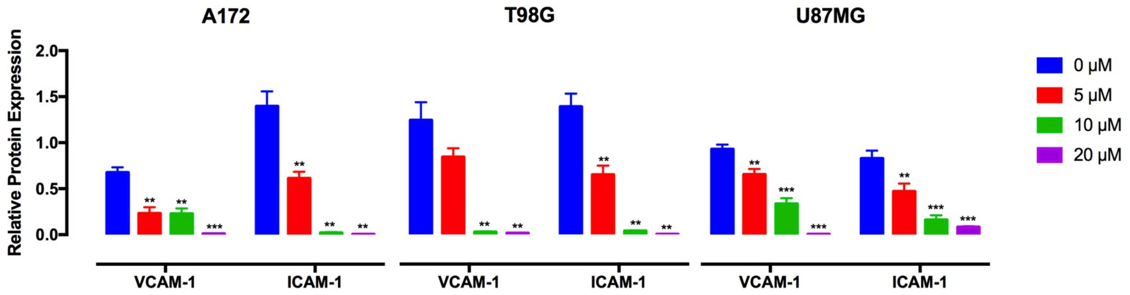

Supplementary Fig. 2

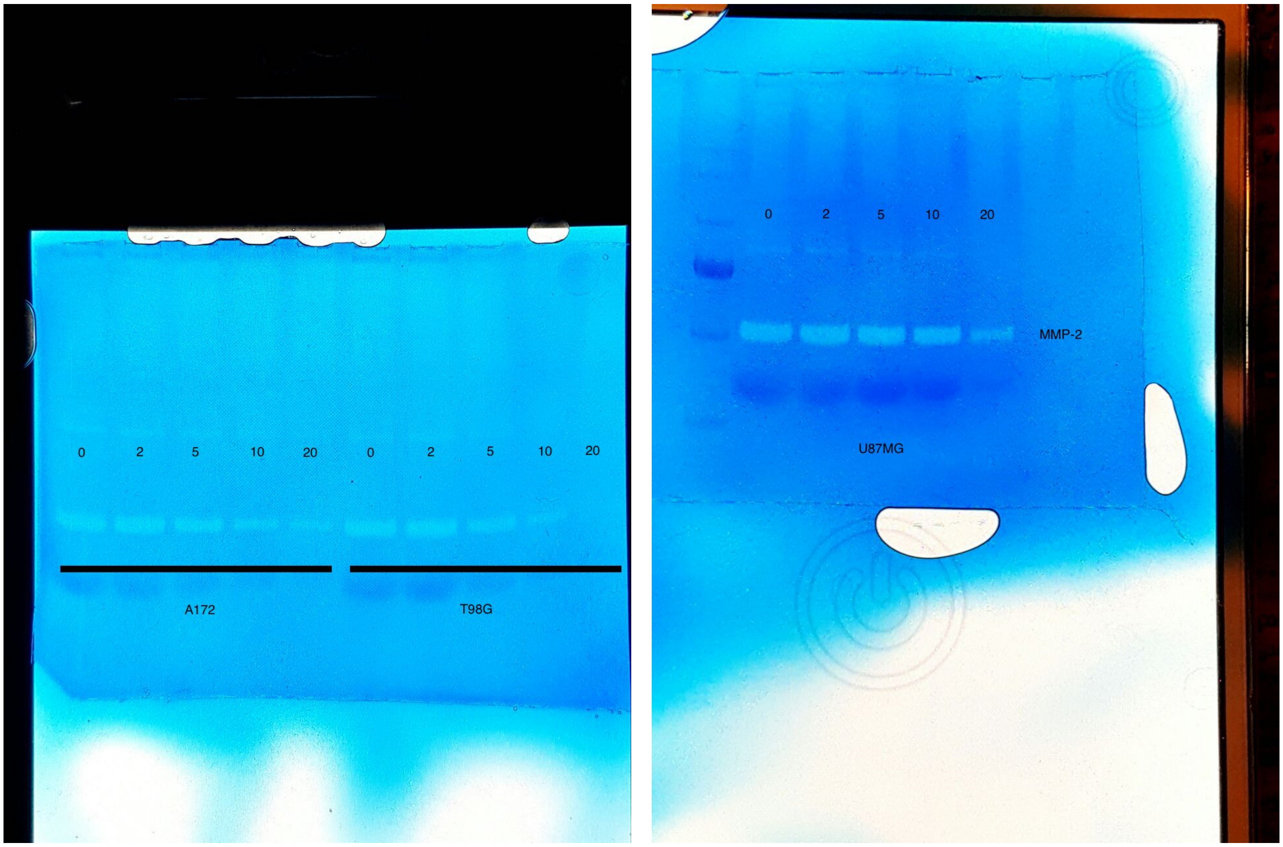

Supplementary Fig. 3

Supplementary Table 1

Concentrations		fa	CI	DRI	
**Tivozanib (μM)**	Irinotecan (μg/mL)			Tivozanib	Irinotecan
		**T98G**			
**10**	0.1	0.5	0.73	1.37	645.91
**10**	0.5	0.5	0.72	1.41	134.43
**10**	1	0.52	0.69	1.48	72.5
**10**	2.5	0.5	0.69	1.51	30.1
**10**	5	0.53	0.69	1.59	16.22
**10**	10	0.54	0.72	1.65	8.65
**10**	25	0.61	0.65	2.17	5.34
		**U87MG**			
**10**	0.1	0.31	1	1	97.77
**10**	0.5	0.34	0.95	1.1	23.41
**10**	1	0.37	0.89	1.22	14.18
**10**	2.5	0.39	0.94	1.29	6.24
**10**	5	0.45	0.87	1.55	4.4
**10**	10	0.56	0.71	2.14	4
**10**	25	0.65	0.73	2.79	2.66

Supplementary Table 2

**Concentrations (µM)**		**fa**	**CI**	**DRI**	
**Tivozanib**	Temozolomide			Tivozanib	Temozolomide
		**T98G**			
**10**	50	0.42	0.97	1.1	16.98
**10**	100	0.44	0.92	1.24	8.92
**10**	200	0.47	0.88	1.5	4.82
**10**	500	0.51	1	1.88	2.12
**10**	1000	0.66	0.85	4.85	1.55
**10**	2500	0.72	1.47	7.61	0.75
		**U87MG**			
**10**	50	0.37	0.69	1.7	9.94
**10**	100	0.39	0.73	1.83	5.34
**10**	200	0.4	0.87	1.94	2.83
**10**	500	0.44	1.1	2.45	1.43
**10**	1000	0.58	0.89	4.96	1.45
**10**	2500	0.73	0.79	12.11	1.42

Supplementary legends

Supplementary Fig. 1: Quantification of protein expression. Quantification of protein band intensities was done using the ImageJ software after normalising to the corresponding β-actin levels. Data are given as mean ± SD. Statistically significant values of $*p < 0.05$, $**p < 0.01$, and $***p < 0.001$ were determined compared with the control.

Supplementary Fig. 2: Quantification of VCAM-1 and ICAM-1 protein expression. Relative protein expression was obtained by normalisation against β-actin. Data are given as mean ± SD. Statistically significant values of $**p < 0.01$ and $***p < 0.001$ were determined compared with the control.

Supplementary Fig. 3: The effects of tivozanib on MMP-2 enzymatic levels. Gelatinolytic activities are visualized as clear bands against the blue background of stained gelatin. The zymograms are representative of three independent experiments with similar results.

Supplementary Table 1: Combination index (CI) and dose reduction index (DRI) of tivozanib and irinotecan combination in T98G and U87MG cells. DRI represents the order of magnitude of dose reduction that is allowed in combination for a given degree of effect as compared with the dose of each drug alone. ‘‘fa’’ denotes fraction affected.

Supplementary Table 2: CI and DRI values of tivozanib and temozolomide combination in T98G and U87MG cells.
